# 

**Published:** 1916-08

**Authors:** 


ESTABLISHED JULY, 18[illegible]

PUBLISHED MONTHLY AT AUSTIN, TE[illegible]

MRS. F. E. DANIEL, - - - Publisher and M[illegible] Editor

Subscription, $1.00 a Year, in Advance. - - - - Single Copies, 10 Cents

Entered at the Postoffice at Austin, Texas, as second class mail matter.

